# Cement-Modified Loess Base for Intercity Railways: Mechanical Strength and Influencing Factors Based on the Vertical Vibration Compaction Method

**DOI:** 10.3390/ma13163643

**Published:** 2020-08-17

**Authors:** Yingjun Jiang, Qilong Li, Yong Yi, Kejia Yuan, Changqing Deng, Tian Tian

**Affiliations:** Key Laboratory for Special Area Highway Engineering of Ministry of Education, Chang’an University, Xi’an 710064, China; jyj@chd.edu.cn (Y.J.); changqingdeng@chd.edu.cn (C.D.); 2019021033@chd.edu.cn (T.T.)

**Keywords:** cement-modified loess, mechanical strength, vertical vibration compaction method, intercity railway subgrade

## Abstract

Cement-modified loess has been used in the recent construction of an intercity high-speed railway in Xi’an, China. This paper studies the mechanical strength of cement-modified loess (CML) compacted by the vertical vibration compaction method (VVCM). First, the reliability of VVCM in compacting CML is evaluated, and then the effects of cement content, compaction coefficient, and curing time on the mechanical strength of CML are analyzed, establishing a strength prediction model. The results show that the correlation of mechanical strength between the CML specimens prepared by VVCM in the laboratory and the core specimens collected on site is as high as 83.8%. The mechanical strength of CML initially show linear growth with increasing cement content and compaction coefficient. The initial growth in CML mechanical strength is followed by a later period, with mechanical strength growth slowing after 28 days. The mechanical strength growth properties of the CML can be accurately predicted via established strength growth equations. The results of this study can guide the design and construction of CML subgrade.

## 1. Introduction

With the rapid development of China’s intercity high-speed railway, quality requirements for the intercity railway subgrade project have increased. High-strength, high-stiffness, uniformly changed subgrade with long-term stability is the prerequisite for ensuring the safe and stable operation of high-speed trains. The Chinese National Standards “Code for Design of Intercity Railway” (TB10623-2014) and “Code for Design of Railway Earth Structure” (TB10001-2016) stipulate that when fine-grained soil fillers are used for the subgrade bed of ballastless track subgrade and, below, the fine-grained soil filler should be improved prior to use [1,2]. Loess is widely distributed along the Xi’an–Hancheng intercity high-speed railway project and has become the main road construction material. Loess is porous and has low bearing capacity and high compressibility; it is also prone to significant settlement after immersion in water [3]. Since loess is a fine-grained soil filler, it needs to be improved with cement. Key to the application of cement-modified loess (CML) is the accurate assessment of its strength characteristics and the factors influencing those characteristics.

Sumesh et al. investigated the mechanical strength of soil mixed with cement and observed that an appropriate amount of cement can effectively improve strength characteristics of soil. [4]. Nilo et al. studied the influence of different cement contents on the unconfined compressive strength (UCS) of cement-modified loess, noting that the UCS of cement soil increases linearly with increasing cement dosage at the same compaction level and the effect of water content on the compressive strength of cement soil is significantly greater than that of the cement dosage [5]. Xing [6], Mohamed [7], and Yong et al. [8] combined scanning electron microscopy with X-ray diffraction to determine the ions affecting the structure and strength of cement-modified soil: Al^3+^, Ca^2+^, Mg^2+^, and others. Correia [9] and Chore et al. [10] used unconfined compressive strength tests and split tensile tests to, respectively, study the compressive and tensile strength characteristics of cement–soil mixed with fiber. Subramaniam et al. performed a series of resonant column tests and cyclic triaxial experiments to study the effects of effective confining pressure, curing stress, cement content, initial clay water content, and curing time on the shear modulus reduction and damping ratio of cement-treated marine clay [11]. Liu et al. performed dynamic triaxial tests on cement and lime-modified soil blended at various ratios. Their results reveal that, after repeated freeze–thaw cycles, the performance of the modified soil was better than before the modification, and the cement-modified soil performance was superior to that of lime-modified soil [12]. Xiao et al. used cement to treat mixed fill, studied the influence of curing time and cement dosage on the compressive strength of cement soil by controlling the water–cement ratio, and established a single-factor compressive strength prediction equation [13]. Li observed that the mechanical properties of CML gradually improve with increasing cement dosage and denoted that the compressive strength of modified loess with a cement dosage of 3% was greater than 0.35 MPa, meeting the design requirements for the strength of the bottom filler for high-speed railway foundation [14]. Combining the construction practice and indoor test results of railway subgrade engineering in collapsible loess areas, Chen suggested that a 4% cement dosage should be used for the improved loess filler at the bottom of the subgrade bed [15]. For the Bao-lan Passenger Line foundation project, Fang et al. used unconfined compressive strength and axial split tests to analyze the effect of water content on the mechanical strength of the modified loess with a 5% cement dosage [16].

Most of the above studies are based on the traditional heavy compaction test method (HCTM) and the static pressure compaction method (SPCM) to form specimens. The Chinese standard “Code for Design of Railway Earth Structure” and “Code for Soil Test of Railway Engineering” also use the HCTM to determine the optimal moisture content and maximum dry density and SPCM to form specimens [2,17]. UCS is used in evaluation of the performance of subgrade engineering and is an important parameter for determining the optimal cement dosage. The current heavy-duty compaction standard was established in the early 21st century to be compatible with compaction mechanical performance at that time, with characteristics of simple operation and low equipment cost. However, with the development of modern transportation and construction technology and the deepening of engineering practice, the traditional method has lagged behind actual practice. The optimal moisture content determined by the HCTM is too large, and the maximum dry density too small; the correlation between the strength of specimens formed by SPCM and the actual core strength of the road surface is less than 70% [18]. To solve these problems, Jiang et al. studied the effects of compaction method on the mechanical properties of inorganic binder-stabilized materials [18,19,20,21,22,23]. They verified the reliability of VVCM in the compaction of cement-stabilized materials in the laboratory by optimizing the working parameters of the vertical vibration test equipment (VVTE). Their results show that the correlation between the mechanical strength of the cement-stabilized crushed stone specimens formed by VVCM and the mechanical strength of the on-site core could reach 93%. However, the effects of VVCM on the mechanical properties of CML have yet to be studied.

In addition, due to the widespread distribution of loess in China and its variable technical properties, there are many factors affecting the strength characteristics of CML. Even at a given cement dosage, the engineering characteristics of modified loess fillers in different regions will be different [24]. There are few studies on the influence of the compaction coefficient on the mechanical strength of intercity railway subgrade fillers, preventing engineers from fully grasping the change law of CML subgrade strength characteristics. In addition, the relevant intercity railway subgrade design specifications use only seven-day-saturated UCS to control the cement dosage, and there is no specific requirement for the strength, stability, or durability of the subgrade [1]. A single strength index cannot reflect the roadbed’s multi-functional requirements for CML filler. Empirical values for cement content are often used in engineering practice, even though they may not fully incorporate the strength and water stability of loess subgrade, leading to potential economic waste.

Therefore, in the context of the Xi’an–Hancheng intercity high-speed railway project, this paper first evaluates the applicability of VVCM by testing the mechanical strength of laboratory-formed specimens and on-site core specimens. Next, the effects of VVCM on the mechanical strength of CML, including unconfined compressive strength (UCS) and splitting strength (SS), are systematically studied, leading to construction of a strength prediction equation. The influences of the cement dosage, the compaction coefficient, and the curing time on the mechanical strength of CML are then analyzed. The research results provide a strong theoretical framework for the design of loess subgrade for intercity railways.

## 2. Experimental Design

### 2.1. Materials

The experiment used loess soil samples taken along the Xi’an–Hancheng planned intercity railway. The unconfined compressive strength of plain loess at the optimal water content of 12.6% and compaction coefficient of 0.97 is only 1.35 MPa, while the splitting strength is only 0.24 MPa. P.O 42.5 ordinary Portland cement (Shaanxi Yaobai Special Cement Co., Ltd., Weinan, China) was selected for performance tests based on the China National Standard “Code for Design of Railway Earth Structure” (TB10001-2016). The physical properties of loess are shown in Table 1, while the technical properties of cement are shown in Table 2.

### 2.2. Specimen Preparation Methods

In this test, we formed cylindrical specimens with a diameter of 100 mm and a height of 100 mm. Before forming specimens, the air-dried loess sample should be spread flat in the metal plate, and the calculated amount of water should be evenly sprayed on the loess sample, and then fully stirred with a mixing tool until the mixing is uniform. The cement should be mixed into the loess sample after the loess is wetted. The amount of water that should be added to each loess sample was then calculated according to

(1) $$m_{w}=\left(\frac{m_{0}}{1+0.01\omega_{0}}+\frac{m_{c}}{1+0.01\omega_{c}}\right)\times 0.01\omega^{\prime }-\left(\frac{m_{0}\times 0.01\omega_{0}}{1+0.01\omega_{0}}+\frac{m_{c}\times 0.01\omega_{c}}{1+0.01\omega_{c}}\right)$$

where *m_w_* is the amount of water that should be added to each loess sample, g; *m*_0_ is the quality of air-dried loess sample, g; *m_c_* is the quality of cement, g; *w*_0_ is the water content of air-dried loess sample, percent; *w_c_* is the water content of cement, percent; and *w*′ is the required water content of the specimen, percent.

First, the VVCM was used to compact the CML with various water contents for 140 s. After compaction, the densities of CML specimens of various water contents were tested, and the dry density–water content curve was drawn to determine the maximum dry density (MDD) and the optimal water content (OWC) of the CML. Next, according to the MDD and the OWC, VVCM was used to form CML specimens with the specified compaction coefficient. Finally, the prepared specimens were wrapped with plastic film and placed in a curing room with a humidity of 95% and a temperature of 20 ± 2 °C until the time of testing [25].

**Figure 1:**
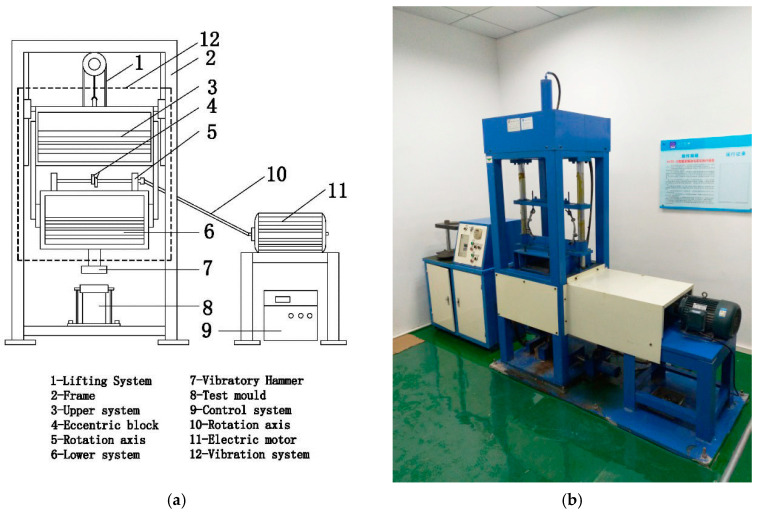
Vertical vibration test equipment (VVTE) schematic: (**a**) diagram of VVTE; and (**b**) photograph of VVTE.

The VVTE parameters are the most important VVCM information (Figure 1) [18,21,22,23,26,27,28]. Its working parameters include a vibration frequency of 35 Hz, nominal amplitude of 1.2 mm, vibration force of 7.6 kN, and upper- and lower-system weights of 120 and 180 kg, respectively.

### 2.3. Test Methods

#### 2.3.1. Unconfined Compressive Strength (UCS) Test

**Figure 2:**
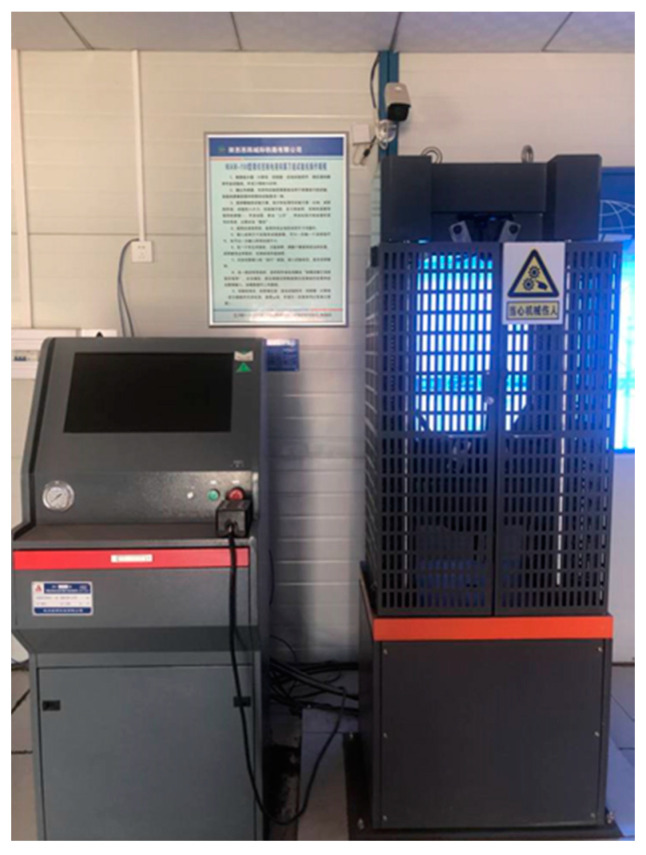
WAW-100 hydraulic servo universal testing machine.

After the specimen reached the curing time, it was removed from the curing room and placed in a water tank with a temperature of 20 ± 2 °C. After 24 h of immersion in water, a WAW-100 hydraulic servo universal testing machine produced by Shanghai Bairuo Testing Instrument Co., Ltd. (Shanghai China) (seeing Figure 2) was used to determine the UCS of the CML specimen, with the loading rate set to 1 mm/min. When the specimen failed, the maximum load P was recorded. The UCS of the sample was then calculated according to

(2) $$q_{u}=\frac{P}{A}$$

where *q_u_* is the UCS of the specimen, MPa; *P* is the maximum load at specimen failure, N; and *A* is the cross-sectional area of the specimen, mm^2^.

#### 2.3.2. Split Strength (SS) Test

After each specimen reached its curing time, it was removed from the curing room and placed in a water tank at a temperature of 20 ± 2 °C. After immersion in water for 24 h, the specimen was placed radially on the WAW-100 test board. The loading rate was set to 1 mm/min until the specimen failed radially, and the maximum load P was recorded. The SS of the specimen was then calculated according to

(3) $$R_{i}=\frac{2P}{\pi dh}$$

where *R_i_* is the SS of the specimen, MPa; *P* is the maximum load at specimen breakage, N; *d* is the diameter of the specimen, mm; and *h* is the height of the specimen after immersion in water, mm.

### 2.4. Reliability Assessment of Vertical Vibration Compaction Method

The key to evaluating test methods is to simulate reality as fully as possible. Therefore, this paper relies on the test section of the Xi’an–Hancheng intercity railway and compares the mechanical strengths of specimens formed by VVCM and SPCM with those of core specimens obtained on site under the same conditions to evaluate the real-world applicability of the VVCM.

The practical CML is compacted with a vibratory roller 4–8 times until the CML reaches compaction coefficients of 0.95, 0.96, and 0.98 in the road construction engineering. In the laboratory, the VVCM and SPCM were used to form specimens with the same compaction coefficients as the on-site core. Afterwards, the specimens were transported to the construction site and maintained with the subgrade on site for the prescribed curing time. After reaching the prescribed curing time, on-site samples were drilled and cored at the engineering site. We chose a uniformly compacted area and used a core-taking machine to take the core. After obtaining core samples, we used a cutting machine to cut core samples into standard sizes. The saturated UCS values of the on-site core and the specimens formed by the VVCM and SPCM were simultaneously tested.

The seven-day (7d) UCS test results and analysis are shown in Table 3; here P_S_/P_X_ is the ratio of the SPCM-formed specimen’s USC value to that of the field core sample, and P_V_/P_X_ is the ratio of the VVCM-formed specimen’s USC value to that of the field core sample.

Table 3 demonstrates that, under a given compaction coefficient and curing conditions, the strength of the core sample on site is consistently higher than those of the lab-formed specimens. In interpreting this result, we note that, when site construction was carried out, the road width was wider than the lab-formed specimens, allowing soil particles to be moved sufficiently. However, the lab specimen formation process was limited by the size of the test mold, so the mobility of the soil particles was diminished, affecting the internal structure [19,20,23,24]. The correlation of UCS values between specimens formed by the VVCM and corresponding on-site core samples was as high as 83.8%, while the same correlation between specimens formed by the SPCM and corresponding on-site core samples was less than 70%. This result indicates that the strengths of specimens formed by VVCM were closer to those of corresponding on-site core samples. It shows that specimens formed by VVCM can simulate field construction and predict the real-world properties of CML more accurately.

### 2.5. Test Plan Design

#### 2.5.1. Influence of Cement Dosage

The hydration and pozzolanic reactions of cement-stabilized clays and the interactions between the hydrates and particles can affect the stabilization process [29]. To study the effect of cement dosage on the mechanical strength of CML, a variety of cement dosages were tested: 2%, 3%, 4%, 6%, and 8%.

#### 2.5.2. Influence of Compaction Coefficient

Chinese national standard TB10001-2016 stipulates that the compaction coefficient of intercity railway subgrade filler be 0.92 or more [2]. The improved performance of modern compaction equipment can further improve the compaction coefficient of loess subgrade [30]. To study the effect of compaction coefficient on the mechanical strength of CML, various compaction coefficients were tested: 0.92, 0.95, 0.97, 1.00, and 1.02.

#### 2.5.3. Influence of Curing Time

Given the changes in CML mechanical properties with the extension of curing time, this study explored the effect of curing time on the mechanical strength of CML and established a mechanical strength prediction equation. For this purpose, various curing times were tested: 7, 14, 28, 60, and 90 days.

## 3. Results and Discussion

### 3.1. Maximum Dry Density and Optimal Water Content

**Figure 3:**
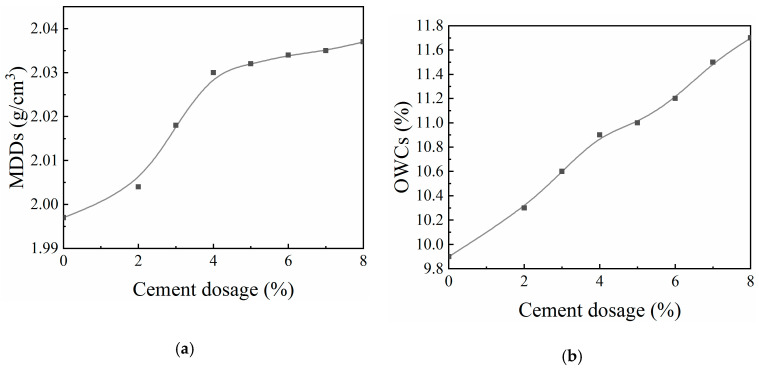
Maximum dry densities (MDDs) and optimal water contents (OWCs) of the cement-modified loess (CML) fabricated using the vertical vibration compaction method (VVCM): (**a**) relationship between cement dosage and MDDs for cement-improved loess; and (**b**) relationship between cement dosage and OWCs for cement-improved loess.

Figure 3 shows the MDD and OWC values of CML specimens with various cement dosages using VVCM compaction for 140 s.

Figure 3 demonstrates that, with increasing cement dosage, the MDD of CML increased. This increase is a simple consequence of to the greater mass per unit volume for cement than for loess. In addition, the cement hydration process made the soil particles more compact, further increasing the dry density. The OWC of CML also increased with increasing cement dosage. This increase is due to the greater quantity of water needed to participate in the chemical reaction for a higher cement dosage.

### 3.2. Mechanical Strength and Strength Prediction Model

#### 3.2.1. Test Results

**Figure 4:**
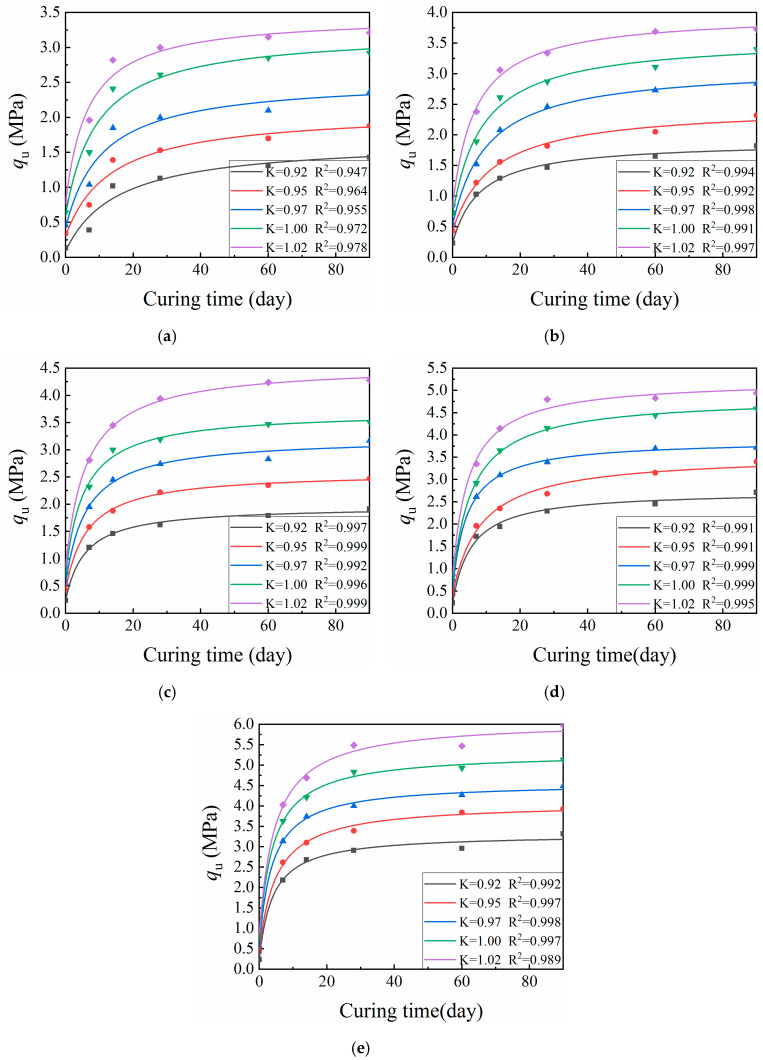
Relationship between age and compressive strength for cement-improved loess: (**a**) cement dosage 2%; (**b**) cement dosage 3%; (**c**) cement dosage 4%; (**d**) cement dosage 6%; and (**e**) cement dosage 8%. K is the compaction coefficient of the specimen.

**Figure 5:**
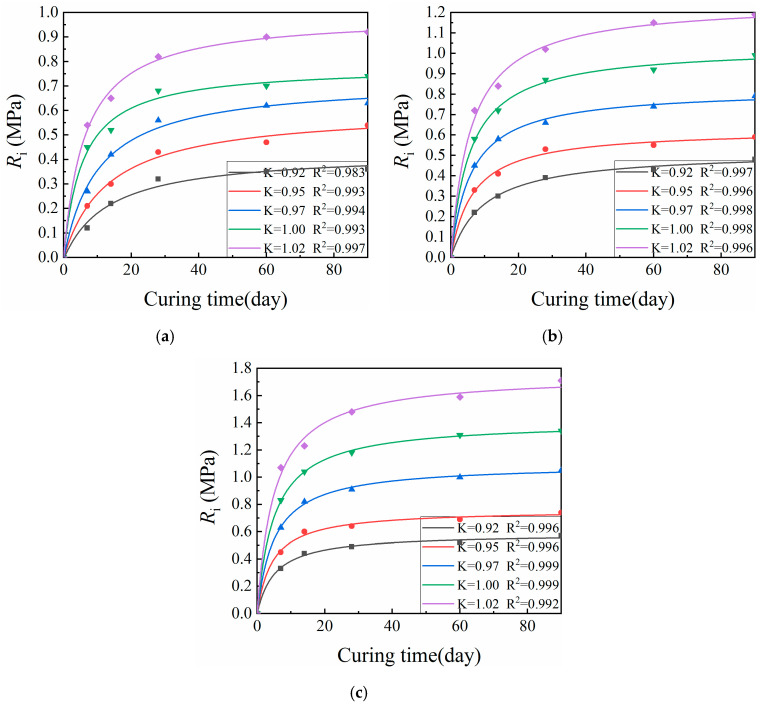
Relationship between age and splitting strength for cement improved loess: (**a**) cement dosage 3%; (**b**) cement dosage 4%; and (**c**) cement dosage 6%. K is the compaction coefficient of the specimen.

Figure 4 shows representative UCS values (*q_u_*) for specimens formed by the VVCM, while Figure 5 shows representative SS values (*R_i_*) for specimens formed by the VVCM. Here, K is the compaction coefficient of the specimen.

Figure 4 and Figure 5 indicate that the relationship of CML mechanical strength with curing time was similar across various cement dosages and compaction coefficients. The mechanical strength growth rate during the first 14 days was significantly greater than afterwards. After 28 days, the growth rate of mechanical strength became slow. The initial CML strength R_0_ was mainly due to physical effects within the material, namely the friction between particles, as well as chemical effects [31,32,33]. With increasing curing time, the cement hydration, coagulation, and hardening reactions continued to proceed, promoting strength growth via consumption of the cement clinker. The rate then gradually slowed down until the clinker was completely consumed, at which point the mechanical strength ceased to increase as the specimen reached its limiting strength *R*_∞_.

In this study, the mechanical strength values at 7 and 28 days were used as examples in the analysis of the effects of cement dosage and compaction coefficient on CML mechanical strength. Compaction coefficients of 0.92 and 0.95 were used as examples in the analysis of the effect of curing time on CML mechanical strength.

#### 3.2.2. Mechanical Strength Prediction Model

The initial strength (*R*_0_) of the CML mainly arises from physical and partial-physicochemical action. With the increase of the curing time, the cement hydration, condensation, and hardening reaction continues, helping to gradually increase the mechanical strength of the CML. Moreover, as the hydration reaction proceeds, the cement clinker is gradually consumed such that the growth of mechanical strength gradually becomes slow. When the cement clinker is exhausted, the mechanical strength no longer increases, i.e., the ultimate strength (*R*_∞_) arising from the physical and physico-chemical action among the cement, water, and loess is obtained [21]. Based on the CML strength growth curve and law established above, this study assumes that a meaningful strength prediction equation meets the following three boundary conditions:

*whenT* = 0, *R*_T_ = *R*_0_;

*whenT* = ∞, *R*_T_ = *R*_∞_;

*R*_0_ < *R*_∞_,

where *T* is the CML curing time, day; *R*_T_ is the CML mechanical strength at curing time *T*, MPa; *R*_0_ is the initial mechanical strength of CML, MPa; and *R*_∞_ is the ultimate mechanical strength of CML, MPa.

Equation (4) [21] is the mechanical strength prediction equation established by the above boundary conditions:

(4) $$R_{T}=R_{\infty }-\frac{R_{\infty }-R_{0}}{\xi T+1}$$

Here, *R*_∞_, *R*_0_, and *ξ* are the regression coefficients of the mechanical strength prediction equation. Initially, *R*_i0_ = 0 for the CML specimens [34,35].

Origin software was used to fit the actual data with Equation (4). Since Equation (4) does not exist in the Origin software, a custom function (Equation (4)) was added and the data were fitted using the added custom function. Subsequently, the mechanical strength prediction equation was obtained. Table 4 and Table 5 provide the regression coefficients and correlation coefficients for the equation parameters.

#### 3.2.3. Verification of Mechanical Strength Prediction Model

To verify the mechanical strength prediction model of CML, we selected CML specimens with a cement dosage of 4% and a compaction coefficient of 0.97 to compare the predicted values and measured values of its UCS and SS. The comparison results are shown in Table 6.

Table 6 shows that the error between the predicted values of UCS obtained from the mechanical strength prediction equation and the actual values of CML does not exceed 6%, and the error between the predicted values of SS and the actual values does not exceed 3%. These results show that the mechanical strength prediction equation established in this study can accurately predict the mechanical strength growth law of the CML.

### 3.3. Factors Influencing Mechanical Strength of Cement-Modified Loess

#### 3.3.1. Cement Dosage

**Figure 6:**
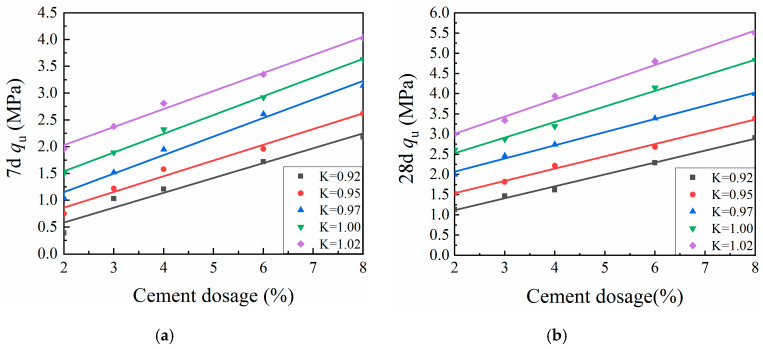
Relationship between cement dosage and unconfined compressive strength (UCS) of cement-modified loess (CML): (**a**) seven-day (7d) UCS; and (**b**) twenty-eight-day (28d) UCS.

**Figure 7:**
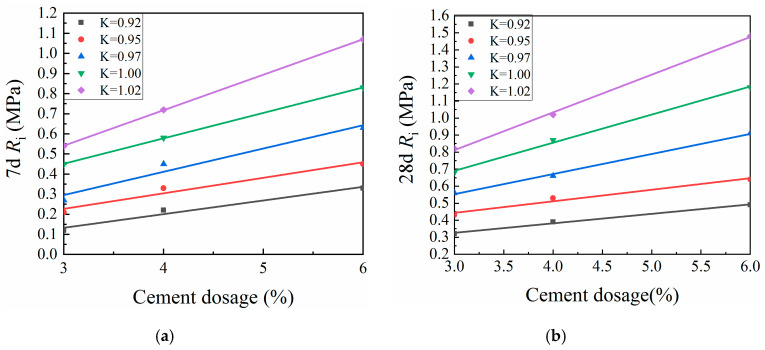
Relationship between cement dosage and splitting strength (SS) of cement-modified loess (CML): (**a**) seven-day (7d) SS; and (**b**) twenty-eight-day (28d) SS.

Figure 6 and Figure 7 show the effects of cement dosage on CML mechanical strength. At a given compaction coefficient and curing time, the unconfined compressive strength and splitting strength of CML gradually increased with increasing cement dosage. For every 1% increase in cement dosage, the UCS of modified loess increased by an average of 21.02% at seven days and 13.08% at 28 days. In addition, the UCS at seven days for a CML specimen with a compaction coefficient of 0.95 and cement content of 4% was similar to that of a specimen with a compaction coefficient of 0.97 and cement content of 3%. At 28 days, the UCS of a CML specimen with a compaction coefficient of 0.95 and cement dosage of 6% was similar to that of a specimen with a compaction coefficient of 0.97 and cement dosage of 3%. These comparisons indicate that, during the CML subgrade filling process, the cement dosage can be appropriately reduced by increasing the compaction coefficient of the subgrade filler. With a cement dosage increase of 1%, the CML splitting strength increased by 14.14–17.88% at seven days and by 10.86–13.47% at 28 days. This finding indicates that the seven-day SS of the specimen at a given compaction coefficient was relatively small and was significantly affected by the cement dosage. With increasing curing time, the effect of cement dosage on splitting strength gradually decreased as the CML stabilized. Therefore, the protection of the roadbed should be strengthened at the initial stage.

#### 3.3.2. Compaction Coefficient

**Figure 8:**
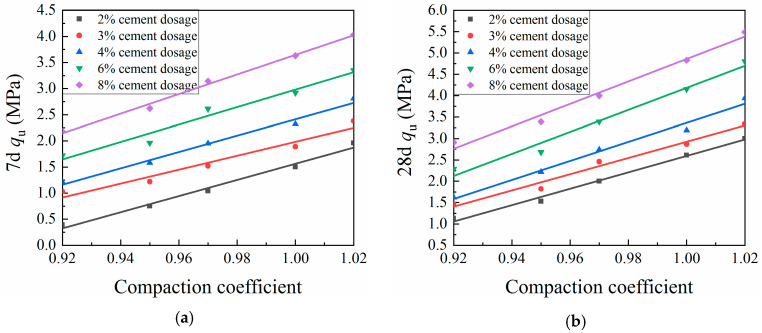
Relationship between compaction coefficient and unconfined compressive strength (UCS) of cement-modified loess (CML): (**a**) seven-day (7d) UCS; and (**b**) twenty-eight-day (28d) UCS.

**Figure 9:**
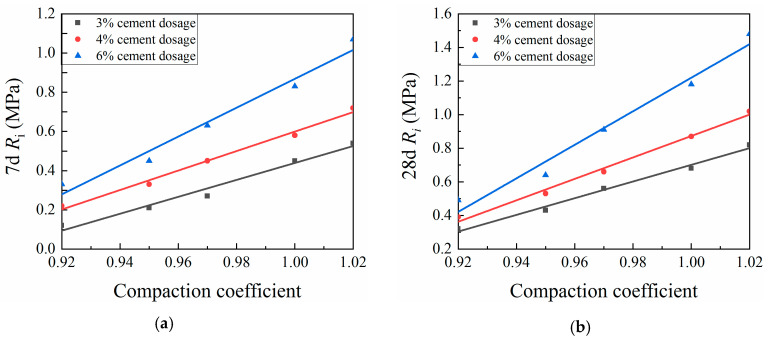
Relationship between compaction coefficient and splitting strength (SS) of cement-modified loess (CML): (**a**) seven-day (7d) SS; and (**b**) twenty-eight-day (28d) SS.

Figure 8 and Figure 9 show the effects of the compaction coefficient on CML mechanical strength. At a given cement dosage, the unconfined compressive strength and splitting strength of CML showed linear growth trends with increasing compaction coefficient. This trend is due to the increase in the dry density of the specimen caused by the increased compaction coefficient, which in turn reduced the internal porosity of the soil. This process brought the soil particles more closely into contact with one another, which increased the resistance to deformation and damage, so that the compressive strength and splitting strength of the specimen were improved [36]. When the compaction coefficient was increased by a 1% increment, the seven-day UCS of the CML with a cement dosage of 2% increased by an average of 12.04%, while the 28-day UCS of the same CML increased by an average of 11.2%. For this same 1% incremental increase in compaction coefficient, the UCS of CML with a cement dosage of 3% or more increased by an average of 8.64% (7 days) and 8.69% (28 days), while the 7- and 28-day SS of the CML increased by averages of 15.44% and 11.78%, respectively. These results indicate that, by increasing the compaction coefficient during the roadbed filling process, the mechanical strength of the CML can be improved to ensure the stability of the roadbed.

#### 3.3.3. Curing Time

**Figure 10:**
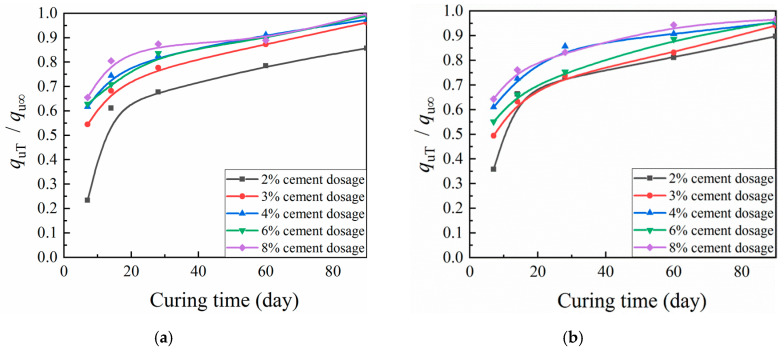
Relationship between *q_u_*_T_/*q_u_*_∞_ and curing time of cement-modified loess (CML): (**a**) compaction coefficient K = 0.92; and (**b**) K = 0.95.

**Figure 11:**
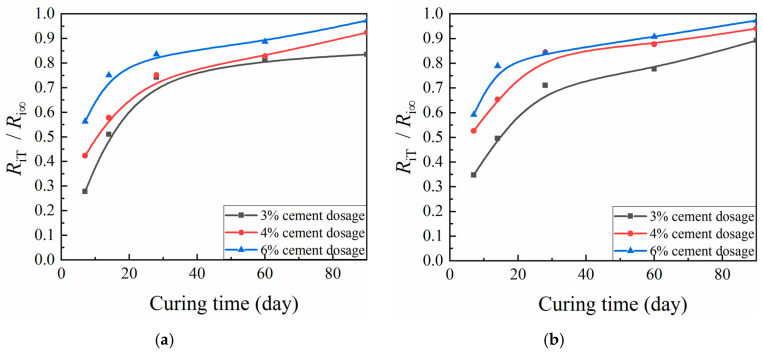
Relationship between *R*_iT_/*R*_i∞_ and curing time of cement-modified loess (CML): (**a**) compaction coefficient K = 0.92; and (**b**) K = 0.95.

According to Figure 10 and Figure 11, similar behavior with respect to curing time was observed across a variety of cement dosages and compaction coefficients for the ratio of CML mechanical strength to corresponding ultimate strength. After a period of explosive growth, the ratio tended to plateau. Mechanical strength predictions suggest a seven-day UCS increase of about 23–66% and a splitting strength increase of about 28–59%. For 28 days, UCS increased by about 68–87%, and splitting strength increased by about 71–84%. At 60 days, UCS increased by about 78–94%, and splitting strength increased by about 78–91%. According to these figures, more attention should be paid to pre-maintenance for CML subgrade.

## 4. Conclusions

VVCM was used to form specimens to study the mechanical strength of CML and the factors that influence those properties. Based on laboratory mechanical tests of CML strength, the following conclusions are drawn:

1. The correlation between the mechanical strength of VVCM molded specimens and on-site core samples was as high as 85.8%, in contrast to the <70% corresponding correlation between SPCM molded specimens and on-site core samples. Clearly, VVCM molded specimens can more accurately simulate field construction and predict CML properties.
2. The unconfined compressive strength and splitting strength of CML showed a linear growth trend with increasing cement dosage and compaction coefficient. During the CML subgrade filling process, the dosage of cement can be appropriately reduced by increasing the subgrade filler’s compaction coefficient.
3. This linear growth trend in CML mechanical strength with increasing cement dosage and compaction coefficient holds for a variety of cement dosages and compaction coefficients. The mechanical strength growth rate in the early stage is significantly greater than in the later stage. Mechanical strength growth tends to slow down after 28 days.

The VVCM developed for the compaction of CML has higher reliability than the traditional SPCM and can accurately reflect the mechanical properties of CML. The influences of various factors on the mechanical strength of CML prepared by VVCM were studied, providing a basis for determining the amount of cement, the compaction coefficient, and the curing time required in any given VVCM engineering application. Two proposed strength prediction models can be used to predict CML mechanical strength for various curing times, thereby reducing the extent of ad hoc experimentation needed. However, the data measured in the laboratory and on-site still mismatch. To make the lab values better match the on-site values, we will propose correction factors through a large number of tests carried out in the future.

## Figures and Tables

**Table 1:** Physical properties of loess.

Particle Density (g/cm^3^)	Liquid Limit (%)	Plastic Limit (%)	Plasticity Index	Percentage Passing (%) of Sieve Sizes (mm)				
				0.25–0.075	0.075–0.05	0.05–0.01	0.01–0.005	≤0.005
2.74	26.4	15.7	10.7	2.47	7.22	53.43	13.83	23.05

**Table 2:** Technical properties of cement.

Test Items	Fineness (%)	Soundness (mm)	Setting Time (min)		Compressive Strength (MPa)		Flexural Strength (MPa)	
			Initial Setting Time	Final Setting Time	3d	28d	3d	28d
test value	2.68	2.05	219	426	14.63	38.99	3.64	8.06
specified value	≤10.0	≤5.0	≥45	≤600	≥11.0	≥32.5	≥2.5	≥5.5

**Table 3:** Seven-day (7d) unconfined compressive strength (UCS) test results for specimens formed by the vertical vibration compaction method (VVCM), specimens formed by the static pressure compaction method (SPCM), and field core samples.

Test Items	Compaction Coefficient	SPCM (MPa)	VVCM (MPa)	On-Site Core Samples (MPa)	P_S_/P_X_ (%) ^1^	P_V_/P_X_ (%) ^2^
7d UCS	0.95	1.38	1.81	2.16	63.9	83.8
	0.96	1.63	2.19	2.64	61.7	83.0
	0.98	2.31	2.86	3.50	66.0	81.7

^1^ P_S_/P_X_ is the ratio of the SPCM-formed specimen’s USC value to that of the field core sample; ^2^ P_V_/P_X_ is the ratio of the VVCM-formed specimen’s USC value to that of the field core sample.

**Table 4:** Estimation equation for unconfined compressive strength of cement-improved loess.

Cement Dosage (%)	Compaction Coefficient	*q_u_*_0_	*q_u_*_∞_	ξ_i_	*R* ^2^
2	0.92	0.134	1.669	0.065	0.947
	0.95	0.340	2.095	0.074	0.964
	0.97	0.460	2.553	0.092	0.955
	1.00	0.643	3.225	0.107	0.972
	1.02	0.803	3.455	0.152	0.978
3	0.92	0.231	1.891	0.123	0.994
	0.95	0.429	2.466	0.085	0.992
	0.97	0.545	3.121	0.098	0.998
	1.00	0.718	3.584	0.113	0.991
	1.02	0.853	3.979	0.149	0.997
4	0.92	0.232	1.962	0.175	0.997
	0.95	0.424	2.590	0.158	0.999
	0.97	0.548	3.226	0.161	0.992
	1.00	0.718	3.708	0.185	0.996
	1.02	0.854	4.560	0.165	0.999
6	0.92	0..232	2.738	0.179	0.991
	0.95	0.423	3.557	0.117	0.991
	0.97	0.558	3.886	0.228	0.999
	1.00	0.722	4.842	0.169	0.999
	1.02	0.855	5.237	0.208	0.995
8	0.92	0.235	3.328	0.251	0.992
	0.95	0.426	4.071	0.201	0.997
	0.97	0.556	4.568	0.258	0.998
	1.00	0.725	5.304	0.245	0.998
	1.02	0.855	6.137	0.208	0.996

**Table 5:** Estimation equation for unconfined splitting strength of cement-improved loess.

Cement Dosage/%	Compaction Coefficient	*R*_i∞_	ξ_i_	*R* ^2^
3	0.92	0.431	0.075	0.983
	0.95	0.605	0.075	0.993
	0.97	0.723	0.098	0.994
	1.00	0.781	0.177	0.993
	1.02	0.988	0.159	0.997
4	0.92	0.519	0.101	0.997
	0.95	0.627	0.153	0.996
	0.97	0.823	0.167	0.998
	1.00	1.033	0.176	0.998
	1.02	1.252	0.170	0.996
6	0.92	0.586	0.192	0.996
	0.95	0.760	0.221	0.996
	0.97	1.095	0.197	0.999
	1.00	1.410	0.201	0.999
	1.02	1.754	0.198	0.995

**Table 6:** The comparison of predicted and measured values of UCS and SS of CML.

Test Items	Curing Time (Day)	Predicted Values (MPa)	Measured Values (MPa)	Error (%)
UCS	7	1.97	1.95	1
	14	2.4	2.45	2
	28	2.74	2.74	0
	60	2.98	2.83	5.3
	90	3.05	3.17	3.8
SS	7	0.44	0.45	2.2
	14	0.58	0.58	0
	28	0.68	0.66	3
	60	0.75	0.74	1.4
	90	0.77	0.79	2.5

## References

[1] National Railway Administration (2014). Code for Design of Intercity Railway (TB 10623-2014).

[2] National Railway Administration (2016). Code for Design of Railway Earth Structure (TB10001-2016).

[3] Gao G. (1996). The distribution and geotechnical properties of loess soils, lateritic soils and clayey soils in China. Eng. Geol..

[4] Sumesh M., Kalita A., Singh B. (2010). An experimental investigation on strength properties of fly ash blended soils treated with cement. J. Environ. Res. Dev..

[5] Consoli N.C., Foppa D., Festugato L., Heineck K.S. (2007). Key parameters for strength control of artificially cemented soils. J. Geotech. Geoenviron. Eng..

[6] Xing H., Xiong F., Zhou F. (2017). Improvement for the strength of salt-rich soft soil reinforced by cement. Mar. Georesour. Geotech..

[7] Mohamed A.M.O. (2000). The role of clay minerals in marly soils on its stability. Eng. Geol..

[8] Yong R.N., Ouhadi V.R. (2007). Experimental study on instability of bases on natural and lime/cement-stabilized clayey soils. Appl. Clay Sci..

[9] Correia A.A.S., Paulo J.V.O., Custodio D.G. (2015). Effect of polypropylene fibers on the compressive and tensile strength of a soft soil, artificially stabilized with binders. Geotext. Geomembr..

[10] Chore H.S., Vaidya M.K. (2015). Streength characterization of fiber reinforced cement-flv ash mixes. Int. J. Geosynth. Ground Eng..

[11] Subramaniam P., Banerjee S. (2020). Dynamic properties of cement-treated marine clay. Int. J. Geomech..

[12] Liu J., Wang T., Tian Y. (2010). Experimental study of the dynamic properties of cement-and lime-modified clay soils subjected to freeze–thaw cycles. Cold Reg. Sci. Technol..

[13] Taoli X. (2007). Analysis of Soft Ground Settlement of Highway and Prediction of Post-Construction Settlement [D].

[14] Shanzhen L. (2017). Research on Dynamic Stability of Loess Roadbed Improved by Cement on High-Speed Railway [D].

[15] Xianghong C. (2014). Research on compaction of moisture-improved loess subgrade cement improved soil filling. Manag. Technol. Small Medium-Sized Enterpr. (Late J.).

[16] Jun F., Qingguo L., Pu H., Lili W. (2018). Comparative experimental study on tensile and compressive strength of Lanzhou cement improved loess. Railw. Constr..

[17] National Railway Administration (2010). Code for Soil Test of Railway Engineering (TB 10102-2010).

[18] Deng C., Jiang Y., Yuan K., Tian T., Yi Y. (2020). Mechanical properties of vertical vibration compacted lime-fly ash-stabilized macadam material. Constr. Build. Mater..

[19] Jiang Y., Fan L. (2013). An investigation of mechanical behavior of cement-stabilized crushed rock material using different compaction methods. Constr. Build. Mater..

[20] Jiang Y., Xue J. (2019). Investigation into physical and mechanical properties of SRX-stabilised crushed rock using different compaction methods. Int. J. Pavement Eng..

[21] Deng C., Jiang Y., Lin H., Ji X. (2019). Mechanical-strength-growth law and predictive model for cement-stabilized macadam. Constr. Build. Mater..

[22] Ji X., Li X., Hou Y., Wang T. (2019). Comparison on properties of cement-stabilised gravel prepared by different laboratory compaction methods. Road Mater. Pavement Des..

[23] Deng C., Jiang Y., Tian T., Chen Z. (2019). Resilient modulus and influencing factors of vertical vibration compacted cement-stabilized macadam. Int. J. Pavement Eng..

[24] Gu K., Chen B. (2020). Loess stabilization using cement, waste phosphogypsum, fly ash and quicklime for self-compacting rammed earth construction. Constr. Build. Mater..

[25] Zheng Z. (2020). Vibration Characteristics and Vertical Vibration of Loess Filling in Intercity Railway Research on Compaction Test Method.

[26] Deng C., Jiang Y., Lin H., Chen Z., Ji X. (2019). Influence of gradations on performance of emulsified asphalt cold recycled mixture produced using vertical vibration compaction method. Road Mater. Pavement Des..

[27] Jiang Y., Deng C., Xue J., Liu H., Chen Z. (2020). Investigation of the fatigue properties of asphalt mixture designed using vertical vibration method. Road Mater. Pavement Des..

[28] Jiang Y., Deng C., Li Q., Liu H. (2019). Effect of compaction methods on physical and mechanical properties of asphalt mixture. J. Mater. Civ. Eng..

[29] Kim Y.T., Do T.H. (2012). Effect of bottom ash particle size on strength development in composite geomaterial. Eng. Geol..

[30] Feng S.J., Du F.L., Shi Z.M., Shui W., Tan K. (2015). Field study on the reinforcement of collapsible loess using dynamic compaction. Eng. Geol..

[31] Lifeng F. (2009). Experimental Study on Improved Loess Using Cement for Subgrade of Zhengzhou-Xi’an Dedicated Passenger Special Line.

[32] Fengming W.U. (2015). Engineering Properties Experimental Study Baolan of Collapsible Loess.

[33] Lorenzo G.A., Bergado D.T. (2004). Fundamental parameters of cement-admixed clay—New approach. J. Geotech. Geoenviron. Eng..

[34] Jiang Y.J., Li M.J., Zhang J.J., Wang S. (2010). Influence factors of strength properties of cement stabilization of crushed aggregate. J. Chang’an Univ. Nat. Sci. Ed..

[35] Yingjun J., Fuyu W., Bin L.I.U. (2009). Research on strength properties of cement stabilization of crushed aggregate. J. Wuhan Univ. Technol..

[36] Hu R.L., Yeung M.R., Lee C.F., Wang S.J. (2001). Mechanical behavior and microstructural variation of loess under dynamic compaction. Eng. Geol..

